# Pre-Transplant Cytokine Levels as Signatures of Microvascular Inflammation in Kidney Allograft Biopsies

**DOI:** 10.7759/cureus.57622

**Published:** 2024-04-04

**Authors:** Priscilla Charles, Srinivas Nagaram, Sreejith Parameswaran, Srinivas BH, Debasis Gochhait, Pragasam Viswanathan, Rajesh Nachiappa Ganesh

**Affiliations:** 1 Pathology, Jawaharlal Institute of Postgraduate Medical Education and Research, Puducherry, IND; 2 Nephrology, Jawaharlal Institute of Postgraduate Medical Education and Research, Puducherry, IND; 3 School of Biosciences and Technology, Vellore Institute of Technology, Vellore, IND

**Keywords:** il-17, tgf-beta, il-6, foxp3, cytokines, kidney allograft, microvascular inflammation

## Abstract

Background: The presence of microvascular inflammation (MVI) characterized by leukocyte margination in the glomeruli (glomerulitis, Banff score ‘g’) and peritubular capillaries (peritubular capillaritis, Banff score ‘ptc’) is a hallmark histological feature of antibody-mediated rejection (AMR), even in the absence of circumferential C4d positivity. In this study, we assessed the efficacy of pre-transplant plasma cytokines as an ancillary screening tool to identify MVI in kidney allograft indication biopsies to facilitate better graft survival.

Method: This single-center prospective analytical study comprises 38 kidney transplant recipients whose peripheral blood was collected before transplant and assessed for the plasma cytokine concentrations of FOXP3, IL-6, TGF beta, and IL-17 using enzyme-linked immunosorbent assays (ELISA). Histopathological assessment was done in post-transplant indication biopsies, and Banff scores of ‘g+ ptc’ were calculated to categorize recipients into three MVI groups. The correlational, regression, and ROC curve analyses were used to assess the association and predictive ability of the cytokines with respect to MVI.

Results: In our study cohort, 27 recipients had MVI=0, five had MVI=1, and six had MVI≥2. A significant difference in plasma cytokines was observed between these groups, and we found a strong negative correlation of FOXP3 with MVI, whereas a strong positive correlation of IL-6, TGF beta, and IL-17 was recorded with MVI. We have also assessed the predictive ability of these cytokines, FOXP3, IL-6, TGF-beta, and IL-17, through the ROC curve, which showed an AUC of 0.70, 0.76, 0.84, and 0.72, respectively.

Conclusion: Our findings suggest that the pre-transplant levels of cytokines FOXP3, IL-6, TGF-beta, and IL-17 could be measured to identify recipients at risk of post-transplant MVI, which could further serve as an additional tool for effective management of the kidney allograft.

## Introduction

Microvascular inflammation (MVI) in kidney biopsies is defined as the leukocyte margination in the glomeruli and peritubular capillaries, represented by the glomerulitis ‘g’ score and the peritubular capillaritis ‘ptc’ score [1]. Since 2013, the Banff group has emphasized the concept of C4d negative antibody-mediated rejection (AMR), and hence the objective assessment of MVI ≥2 as an important feature to diagnose AMR assumes significance [2]. However, it is noteworthy that MVI could also be seen in other conditions like glomerulonephritis, acute T-cell-mediated rejection (TCMR), interstitial nephritis, pyelonephritis, and acute tubular necrosis [3,4]. The advent of molecular techniques has further deciphered the significance of MVI in ABMR and other kidney allograft conditions [5]. Early identification of sub-clinical AMR would require protocol biopsies, which can be confounded by very subtle changes in the histopathology, compound reproducibility in MVI scoring, variable analytical sensitivity of C4d staining, and heterogeneity in donor-specific antibody (DSA) [6]. The objective of this study was to find minimally invasive biomarkers to screen and identify recipients who have post-transplant MVI through the peripheral blood assessment of cytokines at the pre-transplant period, just before the start of induction immunosuppression. By doing so, we may be able to carefully screen and monitor the recipients at frequent intervals for the early identification of MVI in the allograft.

The initiation of inflammation is triggered by various mechanisms through which cytokines play a major role in the induction and effector phases of all immune and inflammatory responses [7]. The TH1 and TH2 responses in kidney transplantation are mainly triggered by the cytokines [8], and hence, a dysregulated production of the proinflammatory or regulatory cytokines is responsible for the kidney diseases post-transplantation, significantly impacting allograft survival. Thus, in this study, we have assessed a set of four cytokines, FOXP3, IL-6, IL-17, and TGF beta, which are known to be involved in the inflammatory and regulatory responses in kidney transplantation. FOXP3 and TGF beta have a pleiotropic role in the regulation of T-helper cells, while IL-17 has a role in the regulation and execution pathways of T-helper 17 cells. IL-6 has a multidimensional role in immune regulation and inflammatory pathways. There is literature on the role of FOXP3, IL-6, IL17, and TGF-beta in influencing graft rejection in transplant settings. To the best of our knowledge, this is the first study to explore the association of plasma cytokines with MVI, which, in turn, aims to serve as a minimally invasive surrogate to identify recipients prone to MVI.

## Materials and methods

Study population

This single-center prospective analytical study was conducted between 2018 and 2020, and all the study participants were followed up for three years from the date of transplant. The study population consisted of 38 consecutive live-related kidney transplant recipients, whose demographic data, clinical information, and 2-ml peripheral blood samples were collected once before induction therapy (pre-transplant) after obtaining the institute’s ethical clearance and informed written consent from the study participants. Deceased donor transplants and transplant recipients with HIV positivity and malignancies were excluded from the study to minimize confounders due to immunosuppression. Protocol graft biopsies are not practiced in our center, and all the patients who had clinically unexplained renal dysfunction underwent indication graft biopsies to identify the cause of the graft injury. Of the 38 recipients, 29 of them had kidney biopsies performed at varying time intervals post-transplant based on their clinical indication. The majority of the graft biopsies in the first-year post-transplant had a high index of suspicion for graft rejection but had concurrent differential diagnoses of calcineurin-induced drug toxicity, atypical bacterial or viral infections, recurrent or de-novo glomerulonephritis, acute tubular, interstitial, or vascular injury due to non-alloimmune insults. In recipients with multiple biopsies, only the one with the highest MVI score was included in the analysis. All research protocols were performed in compliance with Helsinki declaration.

Histopathological evaluation

One core renal biopsy was received in 10% neutral buffered formaldehyde (NBF), normal saline, and 2.5% glutaraldehyde. The tissue was fixed in 10% NBF and processed in a Leica automated tissue processor (Wetzlar, Germany), and 3-micron sections were cut and stained with hematoxylin and eosin (Catalogue no.: GRM236 and GRM423, Himedia, Mumbai, India), periodic acid Schiff (PAS) (Catalogue no.: K078S, Himedia, Mumbai, India), and Masson Trichome stain (Catalogue no.: RM10050, Himedia, Mumbai, India). The tissue core that was transported in normal saline was cut at 5-micron thickness using Leica cryostat (Wetzlar, Germany) and stained with antibodies IgG (Catalogue no: F020202-2, Dako, California, US), IgA (Catalogue no: F020402-2, Dako, California, US), IgM (Catalogue no: F020302-2, Dako, California, US), C3 (Catalogue no: F020102-2, Dako, California, US), C1q (Catalogue no: F025402-2, Dako, California, US), kappa (Catalogue no: F019802, Dako, California, US) and lambda (Catalogue no: F019902-2, Dako, California, US) stains using DAKO mouse monoclonal antibodies tagged with fluorescein isothiocyanate (FITC) in 1:20 dilution. All the immunofluorescence biopsies were interpreted under an Olympus BX53 fluorescence microscope, and all the findings were recorded for photomicrography. The tissue core that was fixed in glutaraldehyde was used for transmission electron microscopic studies as and when required, based on the findings in light microscopy and immunofluorescence. Immunohistochemistry with C4d (Mouse monoclonal antibody clone C4D204, Catalogue No. PM 146, PathnSitu Biotechnologies, Telangana, India) was performed in all transplant biopsies. Transplant biopsies were evaluated independently by three pathologists for various pathologies as per the Banff 2018 transplant classification scores [9]. 

Cytokine assessment

The cytokine assessment was performed on prospectively collected blood samples for all the patients. Recipients who did not undergo renal biopsy were categorized in the MVI=0 group for statistical analysis. Two ml of peripheral blood (n=38) was collected from the recipients before kidney transplant (pre-transplant), and plasma separation was done by centrifuging the blood at 3000 x g for 15 minutes using a refrigerated centrifuge (5430 R, Eppendorf, Hamburg, Germany). Estimation of plasma cytokine concentration was done by sandwich ELISA technique by following the instructions mentioned in the kits FOXP3 (E0692Hu), IL-6 (E0090Hu), TGF-beta (E3051Hu), and IL-17 (E0142Hu) procured from Bioassay Laboratory, China, and by using an Immunowash 1575 ELISA washer and an Imark microplate reader (BioRad, California, USA).

Statistical analysis

The normality of the data was checked through Shapiro-Wilk normality tests, and normally distributed data was represented as mean± standard deviation, whereas non-normally distributed data was represented as median interquartile range. The Kruskal-Wallis test was used for comparing the differences between groups, and post hoc analysis with Bonferroni corrections was used to find out the actual significance between the groups. Spearman rank correlation was done to find the association of cytokines with MVI, and a logistic regression model was constructed to find the classification accuracy of the cytokines. The ROC curve was also constructed to determine the predictive efficacy of the cytokines. IBM Corp. Released 2010. IBM SPSS Statistics for Windows, Version 19.0. Armonk, NY: IBM Corp. was used to perform the statistical analysis, and a p-value less than 0.05 was significant.

## Results

Characteristics of the study population

Of the 38 patients in the study, 29 underwent indication biopsies in the first year post-transplant. The remaining nine patients did not have any renal biopsies, and their MVI scores were assumed to be 0 for statistical analysis. There was no difference in the time interval for post-transplant biopsies between the three groups. The kidney transplant recipients were categorized into three groups based on their microvascular inflammation score: MVI = 0, MVI = 1, and MVI ≥ 2. Out of the 38 recipients, 27 had an MVI score of 0, whereas five had an MVI score of 1, and six had an MVI score of ≥2. In our study population, we have also observed that four of the recipients, whose MVI score was 1, did not experience allograft rejection, whereas all six recipients with MVI≥2 had allograft rejection within 10 months post-transplant. The demographic characteristics between the three groups are summarized in Table 1. Except for the body mass index (BMI), which varied significantly between the three groups, other demographic parameters like recipients’ and donors’ age, cold and warm ischemia time, and blood pressure did not have significant differences between the three groups.

**Table 1:** Demographic characteristics of kidney transplant recipients are represented as the mean ± standard deviation or median (Q1, Q3) MVI = Microvascular inflammation

Parameter	MVI=0 (n=27)	MVI=1 (n=5)	MVI≥2 (n=6)	Kruskal- Wallis P-value
Recipients’ age (in years)	36.81 ± 8.86	38.33 ± 13.50	36 ± 4.74	0.80
Donors’ age (in years)	42.05 ± 11.68	42.33 ± 13.50	49 ± 12.65	0.80
Cold ischemia time (in minutes)	81 (60, 152)	127 (88, 160)	120 (66, 140)	0.26
Warm ischemia time (in minutes)	6.27 ± 2.10	3.42 ± 1.99	6.37 ± 0.99	0.63
Body mass index (Kg/m^2^)	21.60 (16, 31.90)	17.90 (7.50, 23.80)	16.50 (7.50, 20.50)	0.02
Pre-transplant systolic blood pressure (mmHg)	134 (117, 198)	133 (133, 160)	134 (120, 160)	0.81
Pre-transplant diastolic blood pressure (mmHg)	86 (80, 134)	90 (90,90)	90 (80, 100)	0.25
Post-transplant systolic blood pressure (mmHg)	134 (110, 166)	130 (120,130)	136 (125, 148)	0.76
Post-transplant diastolic blood pressure (mmHg)	81 (70, 102)	80 (80,80)	75 (70, 81)	0.06

Association of pre-transplant plasma cytokines with Banff scores of inflammation

Banff inflammatory scores ‘g,’ ‘t,’ ‘i,’ 'v', and ‘ptc’ were assessed to find their correlation with pre-transplant levels of plasma cytokines FOXP3, IL-6, TGF beta, and IL-17 (Table 2). The pre-transplant levels of TGF beta showed a significant positive correlation with Banff scores ‘g,’ ‘t,’ 'i', and ‘ptc’, whereas IL-17 had a significant positive correlation with Banff scores ‘g,’ ‘i,’ 'v', and ‘ptc’. The cytokine FOXP3 had a significant negative correlation with Banff score ‘I’ and IL-6 had a significant positive correlation with Banff score ‘ptc’. 

**Table 2:** Correlation of pre-transplant plasma cytokines with Banff scores of inflammation post-kidney transplant (n=38) g= glomerulitis, t= tubilitis, i= interstitial inflammation, v= intimal arteritis, ptc= peritubular capillaritis, FOXP3= Forkhead box protein P3, IL-6= Interleukin 6, TGF-beta= Transforming growth factor beta, IL-17= Interleukin 17

Banff Scores of inflammations	FOXP3		IL-6		TGF-β		IL-17	
	Correlation coefficient	P-value	Correlation coefficient	P-value	Correlation coefficient	P-value	Correlation coefficient	P-value
Banff score 'g'	-0.27	0.10	0.06	0.71	0.39	0.02	0.34	0.04
Banff score 't'	-0.03	0.88	0.26	0.12	0.33	0.04	0.32	0.05
Banff score 'i'	-0.36	0.03	0.30	0.06	0.57	<0.01	0.48	<0.01
Banff score 'v'	-0.13	0.42	0.25	0.14	0.05	0.76	0.36	0.03
Banff score 'ptc'	-0.30	0.07	0.38	0.02	0.63	<0.01	0.49	<0.01

The concentration of pre-transplant plasma cytokines in microvascular inflammation groups

**Figure 1:**
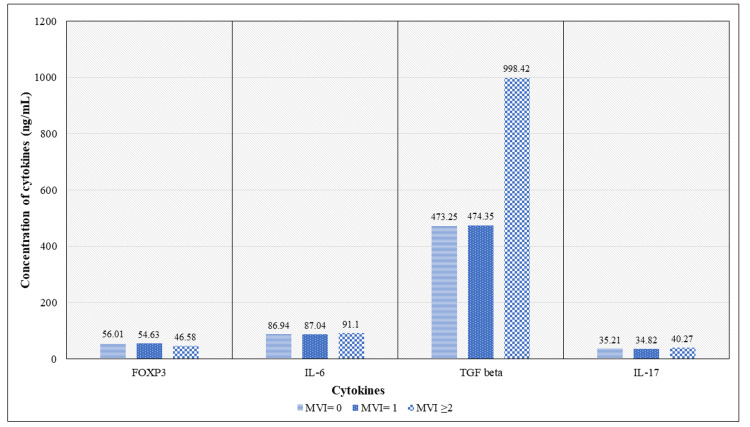
Concentration of pre-transplant plasma cytokines between microvascular inflammation groups MVI = Microvascular inflammation, FOXP3 = Forkhead box protein P3, IL-6 = Interleukin 6, TGF-beta= Transforming growth factor beta, IL-17 = Interleukin 17

The concentration of pre-transplant cytokines between the microvascular inflammation groups was assessed and represented in Figure 1. The Kruskal-Wallis test has shown a significant difference in the concentration of cytokines between the three groups, and post hoc analysis (Table 3) has shown significant differences in the concentration of FOXP3, TGF-beta, and IL-17 between groups MVI=0 and MVI≥2 and between groups MVI=1 and MVI≥2, whereas IL-6 had a significant difference in its concentration only between MVI=1 and MVI≥2. It is important to note that none of the cytokines had a significant difference in their concentrations between MVI = 0 and MVI = 1. 

**Table 3:** Post-hoc analysis showing the median difference of pre-transplant plasma cytokines between the microvascular inflammation groups MVI = Microvascular inflammation, FOXP3= Forkhead box protein P3, IL-6= Interleukin 6, TGF-beta= Transforming growth factor beta, IL-17= Interleukin 17

Microvascular inflammation groups	Mean difference	P-value
FOXP3		
MVI=0 and MVI=1	0.42	1.00
MVI=0 and MVI≥2	7.38	<0.05
MVI=1 and MVI≥2	6.96	<0.05
IL-6		
MVI=0 and MVI=1	3.96	0.11
MVI=0 and MVI≥2	-3.85	0.09
MVI=1 and MVI≥2	-7.81	<0.05
TGF-beta		
MVI=0 and MVI=1	10.37	1.00
MVI=0 and MVI≥2	-409.33	<0.01
MVI=1 and MVI≥2	-4.93	<0.01
IL-17		
MVI=0 and MVI=1	0.39	1.00
MVI=0 and MVI≥2	-4.53	<0.01
MVI=1 and MVI≥2	-4.93	<0.01

Association of pre-transplant cytokines with microvascular inflammation

As there were no significant differences between MVI=0 and MVI=1, and to prevent bias in the analysis due to the smaller sample size in MVI=1 and MVI≥2, we have clubbed together the scores of MVI=1 and MVI≥2 as MVI>0. Spearman correlation analysis was done to assess the relationship between cytokines and MVI groups (MVI = 0 and MVI > 0), in which a strong negative correlation of FOXP3 (r = -0.37, p = 0.02) and a strong positive correlation of IL-6 (r = 0.48, p<0.01), TGF-beta (r = 0.58, p<0.01), and IL-17 (r = 0.43, p = 0.01) was found with MVI scores.

Logistic regression model

**Figure 2:**
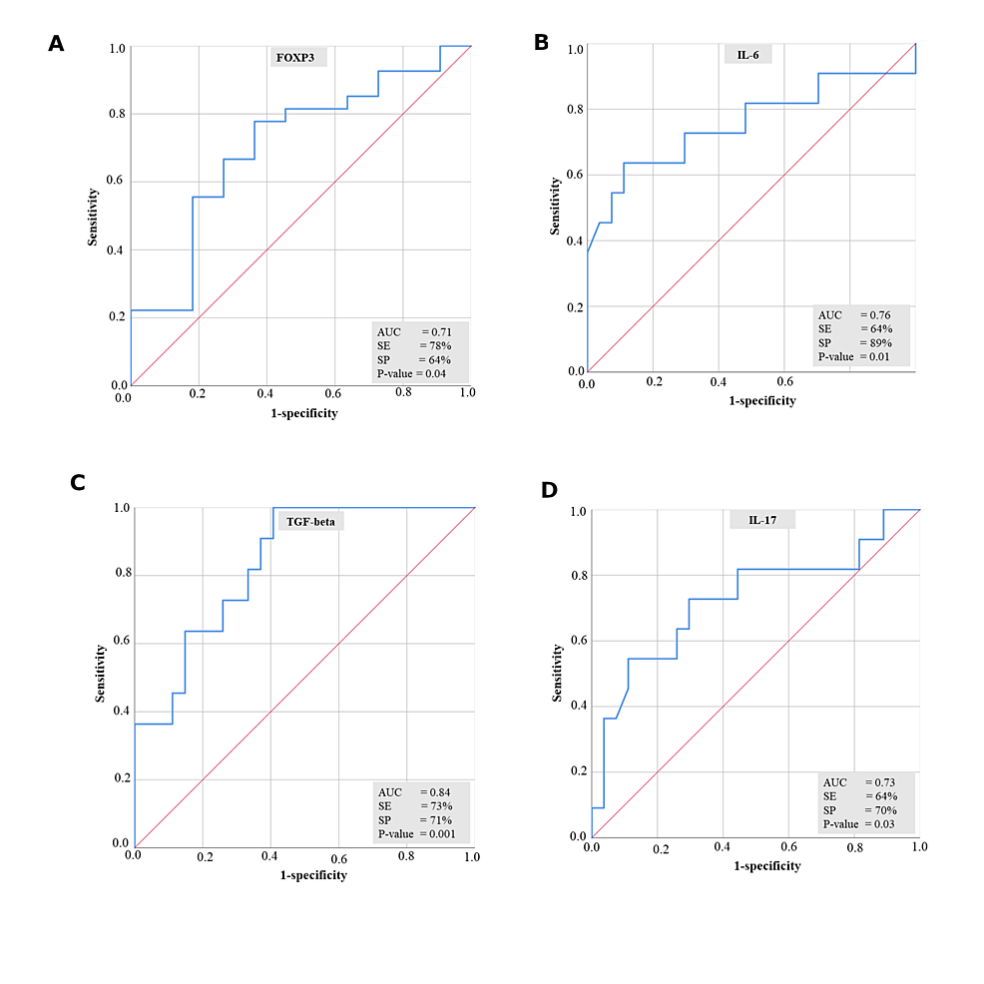
Predictive property of pre-transplant plasma cytokines in MVI. Figure 2a. ROC curve of FOXP3 with respect to MVI. Figure 2b. ROC curve of IL-6 with respect to MVI. Figure 2c. ROC curve of TGF-beta with respect to MVI. Figure 2d. ROC curve of IL-17 with respect to MVI MVI = Microvascular inflammation, FOXP3 = Forkhead box protein P3, IL-6= Interleukin 6, TGF-beta = Transforming growth factor beta, IL-17= Interleukin 17, AUC = Area under the curve, SE = Sensitivity, SP = Specificity

Logistic regression analysis (Table 4) was further used to assess the classification potential of pre-transplant cytokines between the groups (MVI = 0 and MVI > 0), in which the cytokines FOXP3, TGF-beta, and IL-17 had a significant ability to classify between the two groups with a classification accuracy of 71.1, 73.7, and 78.9, respectively. The ROC curve (Figure 2) was constructed based on this logistic regression model, and all the cytokines FOXP3, IL-6, TGF-beta, and IL-17 were significantly able to differentiate between the MVI=0 and MVI>0 groups with an area under the curve (AUC) of 0.71, 0.76, 0.84, and 0.73, respectively.﻿ 

**Table 4:** Correlation of pre-transplant plasma cytokines with microvascular inflammation (g + ptc score) FOXP3 = Forkhead box protein P3, IL-6 = Interleukin 6, TGF-beta = Transforming growth factor beta, IL-17 = Interleukin 17, β0 = intercept, β1 = slope, OR = odds ratio

Cytokines	β_0_	β_1_	Wald statistics ( β_1_)	OR (95% CI)	P-value
FOXP3	7.44	-0.16	4.87	0.85 (0.73, 0.98)	0.03
IL-6	-2.52	0.02	0.04	1.02 (0.85, 1.22)	0.84
TGF-beta	-3.31	0.003	5.46	1.003 (1.001, 1.006)	0.02
IL-17	-14.21	0.37	5.28	1.44 (1.05, 1.97)	0.02

## Discussion

In this study, we aimed to find the association of minimally invasive markers with MVI, and we are to highlight that this is the first novel attempt to evaluate plasma cytokines as signatory molecules for MVI. In general, the MVI scoring in kidney biopsies has challenges in reproducing the diagnosis, which is also proven in a study by Gibson et al., wherein the PTC scoring between six pathologists varied in a fair to moderate range [4], indicating challenges in scoring. Alongside, C4d staining, which was once thought to be a sensitive marker for AMR, is now being marked for its low sensitivity [10], which forced the incorporation of C4d-negative AMR in the Banff 2013 update [2]. Additionally, preformed DSA can cause early rejection and graft loss, whereas de novo formation of DSA that develops post-transplant is associated with late rejections and transplant glomerulopathy [11], resulting in significant morphological heterogeneity in allograft biopsies. Thus, we evaluated cytokine markers to assess the association with MVI, as cytokines are known to have a major role in influencing the immune responses in kidney transplantation [8]. This may potentially be useful as an adjunct for the diagnosis of rejection, along with graft biopsies. A study by Srivastava et al. [12] has previously evaluated plasma biomarkers across diverse kidney diseases and found that sTNFR1, sTNFR2, YKL-40 MCP-1, and suPAR are associated with various kidney diseases like proliferative glomerulonephritis, non-proliferative glomerulopathy, advanced glomerulosclerosis, and diabetic kidney diseases, but the study has not evaluated the plasma biomarkers with MVI in kidney transplant recipients.

In our study, we found that an MVI score of ≥2 was significantly associated with histological evidence of allograft rejection in comparison to an MVI score of ≥1. This is in concordance with a study conducted by Gupta et al. [13], in which acute and chronic AMR were more significant in a group with MVI ≥2.

The major role of cytokines in regulating the inflammatory mechanism progressing to allograft rejection [14] impeded our ability to assess the relationship between plasma cytokines and Banff scores of inflammations. The panel of cytokine markers FOXP3, TGF-beta, IL-6, and IL-17 was found to have a significant association with Banff scores of inflammations, namely ‘g,’ ‘t,’ ‘i,’ 'v', and ‘ptc’. A study by Bunnag et al. [15] recorded a high expression of FOXP3 in renal biopsies with higher interstitial inflammation. In our study, we focused on the pre-transplant levels of plasma cytokines and their correlation with inflammation that occurred post-transplant, making it the first study to note such an association.

Further investigation on the association of plasma cytokines with MVI was made based on our initial assessment, which proved a significant association of plasma cytokines with inflammatory scores. There are very few studies aimed at finding an adjunct ancillary tool for MVI and thus aiding in the diagnosis of AMR. In 2018, Lee et al. [16] conducted a study on 203 kidney transplant recipients to measure the levels of plasma and urinary endocan and reported them as potential biomarkers for MVI. A study by Delsante et al. [6] developed dual IHC staining for CD34 and CD45, which proved to have a higher reproducibility than the conventional light microscopic examination with hematoxylin and eosin and Periodic Acid Schiff stains, thus further improving reproducibility in the identification of MVI. Recently, a multi-center study [17] identified six miRNAs using omics technology and found that the miRNA levels in renal biopsies were in correlation with MVI intensity. To the best of our knowledge, no study has evaluated the pre-transplant levels of plasma cytokines with post-transplant MVI, highlighting the novelty of our study. 

In our study, we found a strong negative correlation between FOXP3 and MVI, wherein the concentration of FOXP3 was higher at MVI = 0 and lower at MVI > 0. In general, FOXP3 has an indispensable role in suppressing inflammation, which is also evident in a study by Saleh et al. [18], where lower levels of FOXP3 mRNA were associated with a prolonged duration of inflammatory responses to stimuli in a group of 507 kidney transplant recipients. IL-6 is generally considered a proinflammatory cytokine, and it acts as a key modulator for T-regulatory cells. Thus, a blockade of IL-6 increases the T-regulatory cells, thereby reducing inflammation in renal allografts [19]. Similarly, in our study, we found a positive correlation between the IL-6 cytokine and MVI, where the concentration of IL-6 was higher in patients with a higher MVI score. In our current finding, we observed that the TGF-beta concentration was higher in patients with a higher MVI score and had a strong positive statistical correlation with MVI. This finding is in contradiction to other reports that state TGF-beta is an anti-inflammatory cytokine that negatively regulates renal inflammation [20]. On the other hand, there is evidence of increased mRNA expression of TGF-beta in the peripheral blood of patients with chronic AMR [21], indirectly validating our result, as MVI is the diagnostic feature of AMR. The IL-17 cytokine has diverse biological roles, like promoting immunity against pathogens as well as driving inflammatory pathology [22]. Likewise, in our study, we found a positive correlation between IL-17 and MVI, further confirming their inflammatory role in kidney transplant recipients.

We have also evaluated the potential of these cytokine markers to identify recipients at risk of post-transplant MVI using a ROC curve to validate our findings. Thus, this study expands our understanding of the role of cytokines as novel signatory molecules for MVI.

A limitation of our study is the lack of assessment of these markers in patients with acute glomerulonephritis, pyelonephritis, or interstitial inflammation, which could also be the primary confounders of this study. Hence, we are unable to say whether these cytokine markers are specifically correlated only with MVI or whether they overlap with inflammation due to any other kidney pathology. Notwithstanding this limitation, we strongly feel that the pre-transplant cytokine levels are useful screening markers to predict MVI in the early post-transplant period. This is because recurrent or de-novo acute proliferative glomerulonephritis causing MVI can be differentiated by a constellation of clinical features and other investigations such as urine microscopy for hematuria, proteinuria, correlation with serum C3 and C4 levels, etc. Similarly, acute pyelonephritis in the post-transplant period can be distinguished by characteristic clinical features such as burning micturition, fever, etc., in addition to urine microscopy for pus cells, neutrophilic leucocytosis, and urine culture. Hence, we propose that our findings may be validated in a larger subset of transplant patients, wherein the potential predictive ability of pre-transplant cytokine levels must be evaluated for diagnostic correlation with MVI in the early one-year post-transplant period. 

## Conclusions

Our study has revealed the significance of plasma cytokines as signature molecules for MVI by elaborating on their association and predictive potential. Our results strongly indicate that the pre-transplant levels of the cytokines FOXP3, IL-6, TGF-beta, and IL-17 can identify recipients at risk of post-transplant MVI. Hence, these plasma markers can potentially serve as a non-invasive adjunct screening tool for the effective management of the kidney allograft.
